# Personals

**Published:** 1918-06

**Authors:** 


### PERSONAL

**Announcement of removal, travel, and other matters of interest are requested. Please report errors in the listing of any physician in the State and other directories, that we may co-operate with the State Society in securing a correct list.**

Dr. Grover Wende of Buffalo is seriously ill with pneumonia. He is now reported to be out of danger.

The Erograph which Dr. Lucien Howe of Buffalo has devised and described for the measurement of fatigue of convergents and accommodations is now being used in the Research Laboratory at Mineola, L. I. Dr. Howe is frequently in Mineola in charge of the use of this instrument.

Dr. Charles Stockton and Dr. Allen Jones attended the convention of the Gastro-Enterologist Society at Atlantic City May 7-8.

Dr. W. H. Bergtold of Denver, a former Buffalonian who graduated at the U. of B. in 1886, has recently published a book on the incubation period of birds. In 1889, he published the first bird list of Erie Co.

Dr. Arthur W. Hurd has resigned as Supt. of the Buffalo State Hospital, a position which he has held for the past 27 years. He will make his future home in California.

Anthony Tall, formerly Supt. of Mt. Sinai Hospital, Cleveland, has been appointed Supt. of the Buffalo Homoeopathic Hospital, in place of Miss Coleman who has resigned.

Dr. Elliott I. Doran of Otisville, has been offered the appointment of Supt. of the new Steuben Co. Tuberculosis Hospital that will soon be opened at Bath.

Dr. Edwin A. Bowerman announces the removal of his office and residence to 375 Linwood Ave., near Utica St.

